# Case control study investigating the clinical utility of NPWT in the perineal region following abdominoperineal resection for rectal adenocarcinoma: a single center study

**DOI:** 10.1186/s12893-022-01746-1

**Published:** 2022-07-30

**Authors:** Tellu Salmenkylä, Katariina Kilpivaara, Pasi Ohtonen, Tero Rautio, Elisa Mäkäräinen

**Affiliations:** 1 Department of Surgery, Medical Research Center, Oulu University Hospital, Oulu, Finland; 2 Research Service Unit, Oulu University Hospital, Oulu, Finland; 3 The Research Unit of Surgery, Anesthesia and Intensive Care, University of Oulu, Oulu, Finland

**Keywords:** Abdominoperineal resection, Incisional negative-pressure wound therapy, Perineal reconstruction, Rectal adenocarcinoma

## Abstract

**Background:**

Perineal wound complications are common after abdominoperineal resection (APR) for rectal adenocarcinoma. Delayed wound healing may postpone postoperative adjuvant therapy and, therefore, lead to a worse survival rate. Negative-pressure wound therapy (NPWT) has been suggested to improve healing, but research on this subject is limited.

**Methods:**

The aim of this study was to assess whether NPWT reduces surgical site infections (SSI) after APR for rectal adenocarcinoma when the closure is performed with a biological mesh and a local flap. A total of 21 consecutive patients had an NPWT device (Avelle, Convatec™) applied to the perineal wound. The study patients were compared to a historical cohort in a case–control setting in relation to age, body mass index, tumor stage, and length of neoadjuvant radiotherapy. The primary outcome was the surgical site infection rate. The secondary outcomes were the wound complication rate, the severity of wound complications measured by the Clavien–Dindo classification, length of hospital stay, and surgical revision rate.

**Results:**

The SSI rate was 33% (7/21) in the NPWT group and 48% (10/21) in the control group, p = 0.55. The overall wound complication rate was 62% (13/21) in NPWT patients and 67% (14/21) in the control group, p > 0.90. The length of hospital stay was 15 days in the NPWT group and 13 in the control group, p = 0.34. The wound severity according to the Clavien–Dindo classification was 3b in 29% (6/21) of the NPWT group and in 38% (8/21) of the control group. A surgical revision had to be performed in 29% (6/21) of the cases in the NPWT group and 38% (8/21) in the control group, p = 0.73.

**Conclusion:**

NPWT did not statistically decrease surgical site infections or reduce wound complication severity in perineal wounds after APR in this case–control study. The results may be explained by technical difficulties in applying NPWT in the perineum, especially in female patients. NPWT devices should be further developed to suit the perineal anatomy before their full effect can be assessed.

*Trial registration* The study was registered as a prospective registry study (266/2018, registered 15th of November 2018)

## Background

Perineal reconstruction after abdominoperineal resection (APR) for rectal adenocarcinoma is associated with a high rate of postoperative surgical site infection (SSI) [1, 2] and delayed wound healing [3]. SSIs cause prolonged hospital stays and delay postoperative adjuvant therapy, thus leading to worse survival rates [4]. Two major risk factors for SSI are receiving neoadjuvant radiotherapy [1–3] and having a body mass index over 30 [2, 5].

The World Health Organization suggests that negative-pressure wound therapy (NPWT) may be used prophylactically on closed high-risk wounds. However, the recommendation is based on limited low quality evidence [6]. Our narrative literature review revealed that NPWT reduces the risk of SSI in closed abdominal incisions [7–10] but may not be effective on deep infections [8] or in reducing rates of dehiscence and seroma [10]. The evidence on using prophylactic NPWT on any wound is limited and more evidence is needed to make solid conclusions on its utility [11].

The aim of this study was to assess whether a NPWT device (Avelle^®^, ConvaTech™) works in preventing wound complications after APR for rectal adenocarcinoma in a prospective case–control setting. Avelle is a topically used NPWT device that functions at negative pressure of 80 mmHg. It consists of a portable pump creating the negative pressure and a self-adhesive dressing that collects the secretions.

## Methods

### Study population

The study was conducted in a case–control setting at Oulu University Hospital. All patients who underwent operations with APR for rectal adenocarcinoma between January 1, 2019 and December 31, 2020 were considered for inclusion. The inclusion criteria were to be aged 18 years or older, have a diagnosis of rectal adenocarcinoma, have undergone APR with curative intent and with the perineal phase operated in a jackknife position, have a life-expectancy of at least a year, and be willing to commit to follow-ups. Emergency procedures and multi-organ resections were excluded. A total of 21 patients filled the inclusion criteria and were selected to the study. The cases were matched with 21 controls from a historical cohort by age, body mass index, tumor stage, and length of neoadjuvant radiotherapy (Table 1).

**Table 1:** Patient characteristics

	NPWT group n = 21	Control group n = 21
Age, mean	71	69
Gender		
Male	19 (90.5)	13 (62)
Female	2 (9.5)	8 (38)
BMI classification		
< 18	0 (0)	0 (0)
18–25	9 (43)	8 (38)
25–30	10 (47.5)	11 (52.5)
> 30	2 (9.5)	2 (9.5)
Neoadjuvant radiation	12 (57)	12 (57)
25 Gy	7 (58)	7 (58)
50 Gy with neoadjuvant chemotherapy	5 (42)	5 (42)
Tumor stage T-stage		
T0	0 (0)	1 (5)
T1	1 (5)	1 (5)
T2	7 (33.5)	6 (28.5)
T3	11 (52)	11 (52)
T4	2 (9.5)	2 (9.5)
Tumor stage N-stage		
0	7 (33)	13 (62)
1		3 (14)
1a	3 (14.5)	
1b	4 (19)	1 (5)
1c	3 (14.5)	1 (5)
2a		3 (14)
2b	4 (19)	
Tumor stage M-stage		
0	20 (95)	19 (90.5)
1	1 (5)	2 (9.5)

Nominal variables reported as counts and percentages (in parentheses)

### Operative technique

The abdominal phase was performed using a total mesorectal excision technique in all study patients. The perineal phase was performed in the jackknife position, as described by Holm et al. [12]. A biological mesh was sutured to the perineal floor using interrupted polydioxanone (PDS™) 0 sutures to form a curved base for the pelvic floor. A local de-epithelialized subcutaneous flap was used to fill the empty space caudal to the mesh. The flap was attached with polyglactin absorbable braided (Vicryl™) 2-0 sutures at the subcutaneous level. A drain with suction was left between the mesh and the flap. Inverted intracutaneous sutures with polyglactin 3-0 (Vicryl™) were used to approximate the wound edges. Intracutaneous absorbable continuous sutures (V-loc90™) were used to close the skin. The NPWT device (Avelle) was attached and sealed with tape that was provided with the device. Negative pressure of 80 mmHg applied.

The NPWT device was intended to be kept on the wound for 7 days. The dressing was changed if the wound secretion was abundant, if there were signs of infection, or if the surgical team wanted to inspect the wound. After a maximum of 7 days, the device and dressing were removed, and wound healing was evaluated. Possible wound complications were evaluated and classified with Clavien–Dindo classification.

### Statistical analysis

Summary statistics are presented as mean with standard deviation unless otherwise stated. A paired samples t-test or the Wilcoxon signed-rank test was used to compare continuous and ordinal scale data, and the McNemar test was used for dichotomous data. Two-tailed p-values are presented. SPSS for Windows (IBM Corp., Armonk, NY, SPSS 26.0, 2019) was used for analyses.

## Results

A total of 21 patients were operated on and received NPWT. Neoadjuvant therapy had been administered to 57% of these patients (Table 1). The SSI rate was 33% (7/21) and 48% (10/21) in the NPWT and the control group, respectively, p = 0.55. The overall wound complication rate was 62% (13/21) in the NPWT group and 67% (14/21) in the control group, p > 0.90. The SSI was graded as Clavien–Dindo 3b and required surgical revision in 29% (6/21) of patients with NPWT and 38% (8/21) in the control group, p = 0.73 (Table 2).

**Table 2:** Outcomes

	NPWT group, n = 21	Control group, n = 21	p-value
Wound complication	13 (62)	14 (67)	> 0.9
Infection	7 (33)	10 (48)	0.55
Seroma	2 (10)	0 (0)	n.d
Dehiscence	2 (10)	1 (5)	> 0.9
Clavien–Dindo classification			
Grade 1	2 (10)	2 (10)	
Grade 2	0 (0)	1 (5)	
Grade 3a	5 (24)	3 (14)	
Grade 3b	6 (29)	8 (38)	0.73
Surgical revision	6 (29)	8 (38)	0.73

Nominal variables reported as counts and percentages (in parentheses). n.d.: Not definable

In the NPWT group, 10% (2/21) of patients had seroma that had to be drained, while no seroma was detected in the control group, p-value not definable. Wound dehiscence occurred in 10% (2/21) of patients in the NPWT group and in 5% (1/21) in the control group, p > 0.9. Length of hospital stay was 15 days in the study group and 13 in the control group, p = 0.34. The NPWT device was removed earlier than the intended 7 days in 62% (13/21) of cases. The device was removed due to vacuum failure in 33% (7/21) of cases and other device-related issues in 29% (6/21) of cases. No infections were detected during the NPWT (Table 3).

**Table 3:** Operative specifications

	NPWT group, n = 21	Control group, n = 21
Technique		
Open	3 (14)	5 (24)
Robotic	9 (43)	9 (43)
Laparoscopic	9 (43)	7 (33)
Drains		
Abdominal drain only	0 (0)	0 (0)
Subcutaneous drain only	4 (19)	14 (67)
Abdominal + subcutaneous drains	17 (81)	6 (28)
None	0 (0)	1 (5)
NPWT device on in days		
1–2	7 (33)	
3–4	1 (5)	
5–6	6 (29)	
7–8	7 (33)	
Reason for removal		
Vacuum failure	7 (33)	
Infection	0 (0)	
As planned	8 (38)	
Other	6 (29)	

Nominal variables reported as counts and percentages (in parentheses)

## Discussion

The use of NPWT failed to demonstrate a decrease in the rate and severity of perineal wound infections. An explanation for this might be the technical difficulties in adhering the NPWT device, as a result of which less than half of the patients had the NPWT device in place for the predetermined 7 days. This study failed to show any advantages in perineal wound healing when using NPWT.

The results of our study should be interpreted in light of its strengths and weaknesses. We collected prospective data for the intervention group, including consecutive operations. The results were compared using a control patient group with identical surgical perineal closures. The study was limited by the small sample size and the retrospective control group. While there was a difference of 15 percentage points in the rate of SSI between the study and control groups, the difference was not statistically significant given the small group size.

Further development of NPWT wound products is required before these devices can be proposed for application to perineal wounds. Adherence to the perineum remains challenging, especially in women, and we speculated whether lining the borders of the devices with adhesive paste would enhance the grip.

## Conclusion

NPWT did not decrease the number of SSIs, severity of surgical site infections, or length of hospital stay in the study patients who underwent APR for rectal adenocarcinoma and perineal reconstruction performed with a biological mesh and a subcutaneous flap. The results may be severely affected by the technical difficulties experienced in applying the NPWT device in the perineal area. Further development of the NPWT device and its dressings is needed. Further research is required to assess the utility of NPWT for perineal wounds.

## Data Availability

The datasets generated and/or analysed during the current study are not publicly available due to Finnish laws on privacy protection but are available from the corresponding author on reasonable request.

## Abbreviations

APR: Abdominoperineal resection
NPWT: Negative-pressure wound therapy
SSI: Surgical site infection
